# Associations between the school food environment, student consumption and body mass index of Canadian adolescents

**DOI:** 10.1186/1479-5868-11-29

**Published:** 2014-03-26

**Authors:** Louise C Mâsse, Judith Evelyn de Niet-Fitzgerald, Allison W Watts, Patti-Jean Naylor, Elizabeth M Saewyc

**Affiliations:** 1 School of Population and Public Health, University of British Columbia, F508-4480 Oak Street, Vancouver, British Columbia V6H 3V4, Canada; 2 School of Exercise Science, Physical and Health Education, University of Victoria, PO Box 3015 STN CSC, Victoria, British Columbia V8W 3P1, Canada; 3 School of Nursing, University of British Columbia, 2211 Wesbrook Mall, Vancouver, British Columbia V6T 2B5, Canada; 4 Department of Pediatrics/School of Population and Public Health, University of British Columbia, F508-4480 Oak Street, Vancouver, BC V6H 3V4, Canada

**Keywords:** School food environment, Diet, Body mass index, School policy, Sugar-sweetened beverages, Adolescents

## Abstract

**Background:**

Increasing attention has been paid to the school food environment as a strategy to reduce childhood obesity. The purpose of this study was to examine associations between the school food environment, students’ dietary intake, and obesity in British Columbia (BC), Canada.

**Methods:**

In 2007/08, principal responses about the school environment (N = 174) were linked to grades 7-12 students (N = 11,385) from corresponding schools, who participated in the BC Adolescent Health Survey. Hierarchical mixed-effect regression analyses examined the association between the school food environment and student’s intake of sugar-sweetened beverages (SSBs), food consumption, and body mass index. Analyses controlled for school setting, neighborhood education level and student’s age and sex.

**Results:**

School availability of SSBs was positively associated with moderate (Odds Ratio (OR) = 1.15, 95% Confidence Interval (CI) = 1.02-1.30) and high (OR = 1.43, 95% CI = 1.13-1.80) SSB intake as were less healthful school nutrition guidelines for moderate SSB consumers only (OR = 0.65, 95% CI = 0.48-0.88). Availability of SSBs at school and its consumption were positively associated with student obesity (OR = 1.50, 95% CI = 1.12-2.01 and OR = 1.66, 95% CI = 1.19-2.34, respectively) but not with overweight. In contrast, consumption of less healthful food was positively associated with overweight (OR = 1.03, 95% CI = 1.01-1.06).

**Conclusions:**

The results of this study provide further evidence to support the important role of schools in shaping adolescents’ dietary habits. Availability and consumption of SSBs, but not less healthful foods, at school were associated with higher adolescent obesity highlighting that other environments also contribute to adolescent obesity.

## Background

Similar to other developed countries, the prevalence of measured overweight and obesity among Canadian youth aged 6 to 17 years in 2010 was 19.5% and 11.6%, respectively [1]. Over consumption of empty calories, defined as calories originating from solid fat and added sugar, is seen as an important contributor to childhood obesity [2]. The consumption of empty calories accounts for about 40% of the total calories consumed by US children (2-18 year) of which 22% are from sugar-sweetened beverages (SSBs) [3]. Similarly, intake of all sugar (natural and added) accounts for 25% of total calories of Canadian adolescents of which 36%-44% are from added sugars (predominantly from SSBs) [4]. Thus public health strategies that promote and enable healthy eating are seen as important investments to address childhood obesity [2,5].

The school food environment is often targeted as children consume roughly 35-47% of their daily dietary intake while at school [6] and schools reach most children across various cultural and socio-demographic backgrounds [7]. In the US, research has shown that students are exposed to a wide variety of less healthful food and beverages while at school [8-10] and are consuming high amounts of less healthful food while at school; including SSBs and energy dense food (pizza, french fries, chips and candies) [6,8,11]. In Canada, there is limited data describing the school food environment, but a recent study from British Columbia (BC) reported that “junk” foods (e.g., pop, cookies, chips, candies) were widely available in middle and high schools through vending machines, cafeterias, tuck shops, and school fundraisers [12]. Furthermore, studies have found that the availability of particular food or beverages at school is associated with consumption of those same items [9,13]. These findings suggest that improvements to the school food environment may enable students to make healthier food choices and lower their body mass index (BMI).

Several studies have reported that school policies, practices, and nutritional capacity and resources restrict the availability of less healthful food and beverages at school [14-17] and improve student dietary intake (e.g., increased fruit consumption and decreased intake of low-nutrient energy dense food) [18,19]. In addition, a large-scale longitudinal evaluation of nutrition policies in US schools found that middle and high school students in states with stronger school nutrition policies gained fewer BMI units and were less likely to remain overweight or obese over time, based on measured BMI [20]. In contrast, other studies have found no relationship between school nutrition policies, practices, or resources with availability or dietary intake, including inconsistent findings for elementary vs. middle/high schools [9,10,16]. Despite mixed results, targeting the school food environment appears promising for addressing obesity globally. As policies are increasingly being used to improve the school food environment, there is a need to gain a clearer understanding of the role of the school food environment on student eating behaviors and BMI.

This study examined the extent to which the school food environment of grades 7 to 12 students in BC, Canada was associated with consumption of SSBs, specific food items and BMI. It was hypothesized that schools with more healthful nutritional environments (e.g. stronger policies, more restriction of unhealthy foods) would have students who consumed fewer SSBs and more healthful food items and have lower BMIs.

## Methods

### Data sources

In the 2007/08 school year, students in grades 7 to 12 completed the BC Adolescent Health Survey (AHS) administered by the McCreary Centre Society every 5^th^ year to monitor the health of BC youth [21]. Of the 283,120 eligible students (eliminating students in non-participating districts (9 out 59) and non-public schools), a random sample of 44,104 students in 463 schools and 1760 classrooms stratified by grades and classes were recruited. In total, 29,315 students completed the survey after incomplete and unusable data were eliminated (66% response rate). The sampling frame ensured a representative sample of BC public school students from grades 7 to 12 (for further details see [21]).

During the same year, public school principals from elementary, middle, and high schools in BC, Canada were invited to complete a nutritional and physical activity school environment survey. In total, 43 of the 59 school districts (73% response rate) provided approval for the study; however, three districts were excluded as they participated in another study conducted by our team. Among schools with students in grades 7 or higher, the school environment survey was completed by 380 principals (48% response rate). For this paper, school and student level data were linked resulting in an analytic sample of 174 schools (67 middle schools, 105 high schools, and two kindergarten to grade 12 schools from 36 districts) and 11,385 students.

### Procedures

All data collection procedures received ethics approval from the University of British Columbia and University of Victoria Research Ethics Board and from school districts.

#### *School data collection*

In January of 2008, school principals were invited to complete the school environment survey (with a pre-paid return envelope). To increase participation, a second mailing and a reminder email with an online link to the consent form and survey were sent. Principals primarily filled out the 30-minute survey but were encouraged to seek the expertise of their nutrition staff to accurately answer sections of the survey. Principals received a nominal incentive ($10 CDN gift card).

#### *Student data collection*

From February to June 2008, students completed the AHS survey. In about half of school districts, parental consent was required, and students in selected classrooms received a consent form to bring home; only students who returned the signed consent form were allowed to complete the survey. In other districts, parental notification with student consent was required, and students within selected classrooms received a notification letter for parents and one for themselves, inviting them to complete the AHS. To preserve anonymity, students were not asked to sign consent forms; instead, completion of the survey indicated their consent or assent. The 45-minute survey was completed during classroom time, for further details see [21].

### Measures

#### *School nutrition environment survey*

The school environment survey integrated five constructs from the Theories of Organizational Changes and Stillman’s Tobacco Policy Framework [22] (adapted for obesity prevention) to measure the school food environment. The psychometric properties of the survey have previously been published [16]. The survey included assessment of: [1] **District policy institutionalization** – a 3-item scale assessing perceived strength of district guidelines with respect to the types of food and beverages made available at school and requirements for nutritional staff and education (α = .79) [16]. Response options were above average, average, and below average; [2] **School food guidelines** – a 7-item scale assessing whether the school had guidelines for; advertising of food/beverage, rewarding with food, subsidizing healthier food/beverage items and requirements for staff and student education (α = .64) [16]. All response options were dichotomized as “yes”/“no” for the analyses; [3] **Nutritional resources** – a 5-item scale modeled after Hoy’s school organization inventory [23] assessed perceived adequacy of nutritional resources compared to other schools in terms of food service staff, food service facilities, access to nutritional expertise, catering options, and food/beverage offerings at school (α = .72) [16]. Response options were above average, average, and below average; [4] **Program participation** – the answers to two “yes”/“no” items assessing participation in the BC Milk Program or the BC School Fruit and Vegetable Nutrition Program were combined to create a 1-item index with participation denoted as none, 1 program, and 2 programs; [16] and [5] **Internal and external support** - a 7-item scale measuring perceived support from the school community (parents, staff, and students) for enacting stricter nutritional guidelines and whether they themselves perceived schools to play a role in obesity prevention (α = .72) [16]. Response options were strongly agree, agree, disagree, and strongly disagree.

Availability of SSBs and food at school was measured with the School Health Policies and Programs Study (SHPPS) questions that assessed food availability in schools (fruit; vegetables; cookies, crackers, cakes, pastries not low in fat; chocolate candy; pizza, hamburgers, or hot dogs; French fried potatoes; salty snacks not low in fat such as regular potato chips and cheese puffs) [24]. We computed a Food Availability Index using the Rideout scoring approach [12] as it takes into account the proportion of healthful to less healthful food items offered at school. The index combined the seven items into 9 possible groups denoting the extent to which more or less healthful food items are available at school. The index regroups various patterns of availability into specific groups, where 1 denotes that only less healthful food items are offered at school, (i.e., sweet/salty snacks and baked goods that are not low in fat; pizza, hamburgers, or hot dogs; and French fried potatoes) and 9 that only healthful food items are offered (i.e., fruit and vegetables).

#### *Student consumption of SSBs and food*

In the AHS, student consumption of SSBs and specific food items was measured with 5 items that asked about the food or beverages consumed yesterday from the time they got up until they went to bed. Student consumption of SSBs was measured by the item that asked whether they drank “pop/soda” yesterday. Student consumption of food was assessed by four items asking whether they ate marker foods fruit; green salad or vegetables; cookies, cake, donuts, and chocolate bars; and pizza, hot dogs, potato chips, and French fries. Response options to these items were “no”, “yes, once” and “yes, twice or more” and were combined to compute a Food Consumption Index utilizing the same scoring approach as the Food Availability Index (described above), with scores ranging from 1 to 9 (where 1 indicates consumption of only less healthful food yesterday and 9 indicates consumption of only healthful food yesterday).

#### *Student BMI*

As part of the AHS, students self-reported their height and weight, with BMI unit computed as kg/m^2^. Student BMI was categorized based on age and gender as follows: “underweight”, “normal weight”, “overweight” and “obese” using Cole et al. criteria [25].

#### *Census data*

The setting and area-level educational attainment of each school was determined by linking school postal codes with the 2006 Canadian Census. The school setting was computed by regrouping Statistics Canada census areas to identify schools located in: an urban setting (communities with an urban core ≥50,000 and a population ≥ 100,000), a suburban setting (communities with an urban core ≥10,000, and a rural setting (all other communities). Educational attainment was operationalized as the percentage of the population with a high school diploma.

### Statistical analyses

We used hierarchical mixed-effects linear or logistic regressions to account for the nesting of students within schools and districts. To examine associations between the school food environment and student consumption of SSBs, we conducted two hierarchical logistic regression analyses comparing no consumption to consuming one pop/soda yesterday (none vs. once) and comparing no consumption to consuming at least two pops/sodas yesterday (none vs. twice+). We used linear regression to examine the association between the school nutrition environment and the student Food Consumption Index.

We used logistic regression for the BMI analyses comparing first the normal weight students versus the overweight students and second the normal weight students versus the obese students. We combined the underweight and normal weight students because we found no difference in the results. For these analyses, we examined associations with the school nutrition environment (Model 1), with student consumption of SSBs and the Food Consumption Index (Model 2) and with both the school nutrition environment and student consumption of SSBs and the Food Consumption Index entered as independent variables (Model 3).

Measures of neighbourhood-level postsecondary education and school setting were entered as school level covariates, while age and sex were entered as student level covariates in the analyses.

Missing data (6.9% SD = 4.6%) for the independent variables and covariates were all imputed using the Expectation Maximization multiple imputation techniques with five replicates [26]. All analyses were conducted in STATA version 11.2 (StataCorp, Texas, US).

## Results

Descriptive information is provided in Table 1. Students were on average 15 years old, equally split by sex (48.1% boys) and 12.7% were categorized as overweight and 3.9% obese. In total, 42.3% of students reported consuming a SSB in the previous day and students scored 5.7 on the Food Consumption Index indicating they consumed some fruits or vegetables as well as some less healthful foods (sweets or fast foods items) in the previous day. Overall, 62.1% of schools were in an urban setting. With respect to the school environment, districts were in the process of institutionalizing nutrition policies. In total, 42% of schools reported healthy nutrition practices with average nutritional resources and 55.6% did not participate in any nutritional program. The school community support for stricter nutritional policies was slightly above average ((2.72/4)/100 = 68%). In total, 43.1% of schools indicated that students had access to SSBs. Finally a score of about 5 for the Food Availability Index indicated that students had access to less healthier food but little to no access to fruit and vegetables.

**Table 1:** Characteristics of schools (N = 174) and grade 7-12 students (N = 11,385), British Columbia, Canada

			**% or mean (SD) [range]**
**Student characteristics**			
Age (N = 11375)			14.9 (1.8) [12.0 – 19.0]
Sex (N = 11368)		Male	48.1%
		Female	51.9%
Body Mass Index (BMI) (N = 9363)		Underweight	4.9%
		Normal weight	78.4%
		Overweight	12.7%
		Obesity	3.9%
**Student behavior**			
Sugar-sweetened beverage consumption (N = 10879)		No	57.7%
		1 yesterday	31.9%
		2+ yesterday	10.4%
Food consumption index (N = 10735)			5.7 (2.4) [1.0 – 9.0]
**School socio-demographic characteristics**			
Postsecondary education (N =171)			32.0% (8.0) [14.61 – 56.86]
School setting (N = 174 )		Urban	62.1%
		Suburban	16.1%
		Rural	21.8%
Median family income (N = 171)			$69,006 ($24,216) [0 – $161,725]
**School environment**			
Policy institutionalization – District guidelines (N = 152 )			2.1 (0.4) [1.0 – 3.0]
Policy institutionalization – school nutrition practices (N = 130 )			0.4 (0.2) [0.0 – 1.0]
Capacity & resources	Nutritional resources (N = 151)		1.9 (0.5) [1.0 – 3.0]
	Program participation (N = 144)	None	55.6%
		1 program	28.5%
		2 programs	16.0%
Internal and external support (N = 148)			2.7 (0.4) [1.7 – 3.7]
Sugar-sweetened beverages availability (N = 174)		No	56.9%
		Yes	43.1%
Food availability index (N = 174)			4.7 (2.1) [1.0 – 9.0]

### Association with student consumption

Four variables were significantly associated with the consumption of SSBs (Table 2) – percent of postsecondary education surrounding the school neighborhood, sex, school guidelines, and availability of SSBs at school. Overall, student consumption of SSBs was lower in schools located in communities with higher rates of post-secondary education (Odds Ratio (OR) = 0.89; p = .006 comparing no consumption to one and OR = 0.84; p = .048 comparing no consumption to two+). In any school environment, the odds of being a moderate or high consumer of SSBs was lower for boys than for girls (OR = 0.49; p < .001 comparing no consumption to one and OR = 0.28; p < .001 comparing no consumption to two+). In addition, the odds of a student being a moderate consumer of SSBs was 0.65 times lower (p = .006) in schools that had healthier nutrition guidelines than those without; however there were no effects for high consumers. Finally, the odds of moderate or high consumption of SSBs were higher in schools that reported having SSBs available than in those that did not (OR = 1.15; p = .022 comparing no consumption to one and OR = 1.43; p = .003 comparing no consumption to two+).

**Table 2:** School factors associated with grade 7-12 students’ sugar-sweetened beverage consumption (N = 10879) and food consumption index (N = 10735)

			**Sugar-sweetened beverage**		**Food consumption index (n = 10496)**
			**None versus one (n = 9518)**	**None versus 2+ (n = 7247)**	
			**OR [95% CI], p-value**	**OR [95% CI], p-value**	**b [95% CI], p-value**
Constant			-	-	4.48 [3.59; 5.37], p < .001
**Covariates**					
School postsecondary education			0.89 [0.81–0.96], p = .01	0.84 [0.71–0.99], p = .048	0.12 [0.01; 0.23], p = .04
School setting		Urban (reference)	1.00	1.00	1.00
		Suburban	1.15 [0.91–1.41], p = .19	1.09 [0.76–1.54], p = .64	0.03 [-0.23; 0.29], p = .83
		Rural	1.01 [0.85–1.21], p = .89	1.11 [0.80–1.52], p = .54	0.04 [-0.21; 0.28], p = .77
Age			0.98 [0.95–1.01], p = .17	1.01 [0.96–1.05], p = .77	0.02 [-0.02; 0.05], p = .33
Sex		Male (reference)	1.00	1.00	1.00
		Female	0.49 [0.45–0.54], p < .001	0.28 [0.24–0.33], p < .001	0.51 [0.42; 0.60], p < .001
**School environment**					
Policy institutionalization – district guidelines			1.08 [0.90–1.28], p = .44	0.98 [0.70–1.35], p = .88	-0.09 [-0.31; 0.14], p = .44
Policy institutionalization – school nutrition practices			0.65 [0.48–0.88], p = .01	0.69 [0.41–1.15], p = .16	0.23 [-0.12; 0.58], p = .19
Capacity & resources	Nutritional resources		1.01 [0.88–1.16], p = .90	0.99 [0.76–1.27], p = .91	-0.06 [-0.24; 0.12], p = .53
	Program participation	None (reference)	1.00	1.00	1.00
		1 program	0.96 [0.84–1.08], p = .48	0.96 [0.73–1.26], p = .77	0.01 [-0.18; 0.20], p = .93
		2 programs	0.97 [0.76–1.23], p = .81	0.88 [0.75–1.26], p = .49	0.07 [-0.15; 0.29], p = .54
Internal and external support			0.91 [0.78–1.06], p = .25	1.08 [0.78–1.48], p = .65	-0.03 [-0.24; 0.19], p = .80
Sugar-sweetened beverages availability		No (reference)	1.00	1.00	NA
		Yes	1.15 [1.02–1.30], p = .02	1.43 [1.13–1.80], p = .003	NA
Food availability index			NA	NA	0.02 [-0.02; 0.05], p = .41

OR = Odds ratio; CI = Confidence Interval; b = non-standardized parameter estimate.

Overall, postsecondary education and sex were the only factors associated with the Food Consumption Index (Table 2). Students attending a school located in a community with higher rates of post-secondary education reported consuming more healthful food (b = 0.12, p = .04). In addition, girls had a healthier Food Consumption Index score than boys (b = 0.51, p < .001).

### Association with student BMI

The findings that examined associations with student BMI are shown in Table 3 for comparisons between normal versus overweight and in Table 4 for comparisons between normal versus obese.

**Table 3:** School factors and grade 7-12 students’ food/beverage consumption associated with Body Mass Index (normal versus overweight) (N = 8995)

			**Model 1 (n = 8834)**	**Model 2 (n = 8361)**	**Model 3 (n = 8209)**
			**OR [95% CI], p-value**	**OR [95% CI], p-value**	**OR [95% CI], p-value**
**Covariates**					
School postsecondary education			0.90 [0.79–1.02], p = .09	0.92 [0.80–1.04], p = .19	0.90 [0.79–1.03], p = .11
School setting		Urban (reference)	1.00	1.00	1.00
		Suburban	1.41 [1.13–1.77], p = .003	1.45 [1.13–1.86], p = .003	1.37 [1.07–1.73], p = .01
		Rural	1.41 [1.14–1.73], p = .002	1.44 [1.14–1.82], p = .003	1.41 [1.13–1.77], p = .003
Age			1.03 [0.99–1.07], p = .17	1.04 [0.99–1.07], p = .09	1.04 [1.00–1.08], p = .05
Sex		Male (reference)	1.00	1.00	1.00
		Female	0.47 [0.41–0.53], p < .001	0.47 [0.41–0.53], p < .001	0.46 [0.41–0.53], p < .001
**School environment**					
Policy institutionalization – district guidelines			0.92 [0.73–1.15], p = .45	-	0.92 [0.73–1.16], p = .49
Policy institutionalization – school nutrition practices			1.16 [0.79–1.67], p = .46	-	1.23 [0.84–1.80], p = .30
Capacity and resources	Nutritional resources		0.85 [0.72–1.02], p = .08	-	0.85 [0.70–1.02], p = .08
	Program participation	None (reference)	1.00	-	1.00
		1 program	1.03 [0.83–1.28], p = .78	-	1.02 [0.82–1.27], p = .87
		2 programs	1.10 [0.83–1.46], p = .49	-	1.14 [0.84–1.54], p = .37
Internal and external support			0.90 [0.73–1.12], p = .35	-	0.88 [0.71–1.11], p = .28
Sugar-sweetened beverages availability		No (reference)	1.00	-	1.00
		Yes	1.16 [0.97–1.39], p = .10	-	1.13 [0.94–1.36], p = .20
Food availability index			1.00 [0.96–1.04], p = .99	-	1.00 [0.96–1.04], p = .86
**Student consumption**					
Sugar-sweetened beverage consumption		None (reference)	-	1.00	1.00
		1 yesterday	-	1.13 [0.98–1.31], p = .10	1.13 [0.97–1.31], p = .12
		2+ yesterday	-	1.12 [0.90–1.39], p = .32	1.13 [0.90–1.40], p = .29
Food consumption index			-	1.03 [1.00–1.06], p = .02	1.03 [1.01–1.06], p = .02

OR = Odds Ratio; CI = Confidence Interval.

**Table 4:** School factors and grade 7-12 students’ beverage/food consumption associated with Body Mass Index (normal versus obese) (N = 8172)

			**Model 1 (n = 8018)**	**Model 2 (n = 7604)**	**Model 3 (n = 7458)**
			**OR [95% CI], p-value**	**OR [95% CI], p-value**	**OR [95% CI], p-value**
**Covariates**					
School postsecondary education			1.00 [0.83–1.20], p = .98	1.00 [0.81–1.23], p = .98	0.98 [0.80–1.20], p = .84
School setting		Urban (reference)	1.00	1.00	1.00
		Suburban	1.53 [1.06–2.23], p = .02	1.67 [1.15–2.44], p = .01	1.52 [1.35–2.23], p = .04
		Rural	1.94 [1.40–1.11], p < .001	2.01 [1.39–2.89], p < .001	1.89 [1.03–2.66], p < .001
Age			1.06 [0.99–1.14], p = .09	1.07 [1.00–1.14], p = .06	1.06 [0.99–1.14], p = .10
Sex		Male (reference)	1.00	1.00	1.00
		Female	0.47[0.38–0.59], p < .001	0.51[0.40–0.64], p < .001	0.51[0.40–0.64], p < .001
**School environment**					
Policy institutionalization – district guidelines			0.87 [0.62–1.23], p = .44	-	0.85 [0.59–1.21], p = .36
Policy institutionalization – school nutrition practices			1.23 [0.66–2.29], p = .50	-	1.15 [0.59–2.23], p = .67
Capacity and resources	Nutritional resources		1.03 [0.77–1.36], p = .85	-	0.97 [0.73–1.31], p = .89
	Program participation	None (reference)	1.00	-	1.00
		1 program	1.22 [0.88–1.68], p = .23	-	1.20 [0.86–1.67], p = .27
		2 programs	0.89 [0.52–1.52], p = .65	-	0.99 [0.57–1.72], p = .96
Internal and external support			0.78 [0.56–1.07], p = .13	-	0.74 [0.53–1.04], p = .08
Sugar-sweetened beverages availability		No (reference)	1.00	-	1.00
		Yes	1.58 [1.20–2.10], p = .001	-	1.50 [1.12–2.01], p = .01
Food availability index			1.03 [0.97–1.09], p = .32	-	1.03 [0.97–1.11], p = .29
**Student consumption**					
Sugar-sweetened beverage consumption		None (reference)	-	1.00	1.00
		1 yesterday	-	1.26 [0.98–1.62], p = .07	1.19 [0.92–1.54], p = .18
		2+ yesterday	-	1.69 [1.20–2.36], p = .003	1.66 [1.19–2.34], p = .003
Food consumption index			-	1.04 [0.99–1.08], p = .15	1.03 [0.98–1.08], p = .27

OR = Odds ratio; CI = Confidence Interval.

Student and school demographic factors and student consumption, but not school environment, were significantly associated with the odds of a student being overweight versus normal weight (model 3, Table 3). Students from schools in suburban and rural settings had higher odds of being overweight than those in an urban setting (OR = 1.37, p = .011 and OR = 1.41, p = .003, respectively). In addition, girls had lower odds of being overweight than boys (OR = 0.46, p < .001) and students who reported consuming less healthful foods had higher odds of being overweight (OR = 1.03, p = .020).

Results comparing obese versus normal weight students (Model 3, Table 4) indicate that the school setting, sex, availability of SSBs at school, and student consumption of SSBs were significantly associated with the odds of a student being obese versus normal weight. Overall, students in suburban and rural schools had greater odds of being obese than those who attended a school in an urban setting (OR = 1.52, p = .035 and OR = 1.89, p < .001, respectively). Girls had lower odds of being obese than boys (OR = 0.51, p = <.001). Notably, students had greater odds of being obese than normal weight in schools where SSBs were readily available (OR = 1.50, p = .007) and if they reported consuming more than one SSB in the previous day (OR = 1.66, p = .003).

## Discussion

This study comprehensively examined associations between the school food environment and student consumption, and in turn, associations with BMI. We found that the availability of SSBs and nutritional practices at school were associated with consumption of SSBs, but no associations with consumption of other less healthful foods. We also found that both the availability of SSBs and less healthful foods were associated with student BMI; although these associations differed by BMI category (overweight versus obese). Specifically, the association with SSBs was only observed among the obese adolescents, whereas an association with less healthful foods was only observed among the overweight adolescents. Our findings add to the limited and inconsistent findings in this area [27] and provide further support for improving the school food environment to reduce childhood obesity.

Similar to previous studies [18,19,28], the availability of SSBs and less healthful nutritional practices at school were both associated with greater SSB consumption highlighting the importance of schools in promoting healthy dietary habits. In contrast, Taber et al. reported that reduced access to SSBs at school reduced purchasing but not overall consumption of SSB [29]. Unlike previous studies [18,19,28,30], associations in this study were observed for SSB consumption only and no associations were found between the other school food environment variables (policies, programs, resources, support, availability) and the Food Consumption Index. Our findings may indicate a need for attention to specificity in food environment measures or reflect limitations in how the Food Consumption and Food Availability Indices were measured.

Access to SSBs at school and their consumption were both associated with obesity providing further support for targeting schools to help address adolescent obesity [20,31]. While the association between SSB consumption and BMI is supported by a recent review [32], the association was present for obese but not overweight adolescents in our study. It is possible that access to SSBs in the school setting may disproportionately affect students who come from a less healthy home environment as they likely consume SSBs both at home and school.

Interestingly, we found an association with our Food Consumption Index and weight, but only for overweight adolescents compared to normal weight adolescents. This association was somewhat expected as a review by Perez-Escamilla [33] supports an association between energy density and BMI in children and adolescents; however, it is somewhat inconsistent that we observed this relationship only among those who were overweight and that consumption of SSBs appeared most related to BMI among obese adolescents. This difference may relate to the limitations of our methodology and is discussed further below.

With respect to the covariates we included in our model, similar to other studies boys were more likely to consume SSBs [34,35], consume energy dense foods [36], and to be overweight and obese than girls [1,35]. Boys are thus a vulnerable group that may benefit from changes to the school food environment to a greater extent than girls might. Similar to other studies, we found that adolescents from more disadvantaged neighborhoods also consumed more SSBs [34,35] and energy dense food [6] and that students in suburban and rural schools were more likely to be overweight and obese [37,38]. These findings highlight groups of adolescents who may be differentially affected by creating healthier eating environments at school.

Our findings should be interpreted in light of the context in which the data were collected. Unlike the US, Canada does not have a national breakfast or school lunch program that is subsidized by the federal government [39]. In BC, while subsidies for school lunch targeting students in need can be obtained from the provincial government, guidelines to regulate the school food environment were first written in 2005, but full implementation was only expected in 2008, after the data were collected for this paper. Interestingly, even in such a varied context and with a guideline in place (albeit without an accountability mechanism) many of our findings echo what others have found in the US [28,30]. Given that the school food environment of Canadian schools was quite different than that of US schools, it remains important to understand whether research from the US context translates into other jurisdictional contexts.

Finally, the study limitations are important to consider when interpreting our results. First, the cross-sectional nature of the data limits our ability to make causal inferences. Second, we used self-report to measure student consumption and BMI, and although commonly used in large studies, are known to be associated with measurement errors that can mask or dampen existing associations. Third, many principals did not complete the survey and half the schools required written parental consent resulting in lower student participation; therefore, we do not know how non-response bias may have affected the results. Fourth, although we evaluated the psychometric properties of our school food environment measures, they were developed or adapted from other measures to fit the BC context. Fifth, we utilized an established measure for the availability of food and beverages at school; [24] however, the measure did not identify if healthier versions of specific food were offered. An unpublished government review suggests that little change in the school food environment had occurred before the full implementation of the first food guidelines were expected in schools which was after we collected the data. Sixth, we highlighted any effects that were significant at a p < .05 but some effects might be less stable as they were not significant at a p < .01 as well (i.e., the association between less healthful foods and overweight adolescents). Given the exploratory nature of our analyses, all of our findings should be replicated. Seventh, each province and territory in Canada has different policies/guidelines affecting the food environment of public schools, and without a federally subsidized school breakfast/lunch program, the generalizability of our findings to other jurisdictions is limited. Finally, measuring consumption over the entire day limits our ability to determine associations with school-specific consumption. This is important as recent data among US children showed a shift in the amount of energy intake obtained from school sources to fast food places [40]. A better understanding of where students’ food purchases occur may shed further light on these findings.

## Conclusions

The results of this study provide further evidence to support the important role of schools in shaping adolescents’ dietary habits. Availability of SSBs at school increased students’ odds of consuming SSBs and being obese and availability of less healthful foods was associated with higher consumption. Creating school environments that are more conducive to healthy eating and implementing a comprehensive approach that includes all of the environments in which adolescents spend their time will likely provide the greatest benefit in supporting healthy food choices and healthy weights.

## Competing interests

The authors declare that they have no competing interests.

## Authors’ contributions

LCM contributed to the conception and design of the study, interpretation of findings, and writing/reviewing the manuscript. JEDNF contributed to the data analysis, interpretation of the findings and writing/reviewing the manuscript. AWW contributed to the interpretation of findings and writing/reviewing the manuscript. PJN contributed to the conception and design of the study, interpretation of findings and reviewing the manuscript. EMS contributed to the conception and design of the study, interpretation of findings and reviewing the manuscript. All authors read and approved the final manuscript.
